# A Comparative Study of Laser-Induced Graphene by CO_2_ Infrared Laser and 355 nm Ultraviolet (UV) Laser

**DOI:** 10.3390/mi11121094

**Published:** 2020-12-11

**Authors:** Liyong Wang, Zhiwen Wang, Ali Naderi Bakhtiyari, Hongyu Zheng

**Affiliations:** Centre for Advanced Laser Manufacturing (CALM), School of Mechanical Engineering, Shandong University of Technology, Zibo 255000, Shandong, China; wly1565983597@163.com (L.W.); wangzw226@163.com (Z.W.); ali.naderi.bakhtiyari@outlook.com (A.N.B.)

**Keywords:** laser-induced graphene (LIG), surface morphologies, polyimide

## Abstract

Laser-induced graphene (LIG) is an emerging technique for producing few-layer graphene or graphene-like material that has recently received increasing attention, due to its unique advantages. Subsequently, a variety of lasers and materials have been used to fabricate LIG using this technique. However, there is a lack of understanding of how different lasers (wavelengths) perform differently in the LIG conversion process. In this study, the produced LIG on polyimide (PI) under a locally water-cooled condition using a 10.6 μm CO_2_ infrared laser and a 355 nm ultraviolet (UV) laser are compared. The experimental investigations reveal that under the same UV and CO_2_ laser fluence, the ablation of PI show different results. Surface morphologies with micron-sized and nanometer pores were formed by the UV laser under different laser fluences, whereas micron-sized pores and sheet structure with fewer pores were produced by the CO_2_ laser. Energy dispersive spectrometry and three-dimensional topography characterization indicate that the photochemical effects were also involved in the LIG conversion with UV laser irradiation. It is also observed through experiments that the photothermal effect contributed to the formation of LIG under both lasers, and the LIG formed on PI substrates by the CO_2_ laser showed better quality and fewer layers.

## 1. Introduction

Graphene has broad applications in sensors, batteries, flexible screens, supercapacitors, and solar cells, owing to its outstanding electrical, thermal, and optical properties [1,2]. Epitaxial growth, chemical vapor deposition (CVD), oxidation-reduction, and organic synthesis are conventional methods to fabricate graphene. These methods have some drawbacks, such as high energy usage, low efficiency, high cost, and environmental pollution, which limit their industrial applications [3]. In the pathway of finding an alternative technique with commercialization capability, a CO_2_ laser was used to convert polyimide (PI) to produce laser-induced graphene (LIG) by Lin et al. [4] in 2014 for the first time. 

Laser direct ablation is an accurate fabrication method that modifies the physical and chemical properties of a small part of the material surface [5]. It can be applied to a variety of materials, including ceramics, metals, and polymers. The advantages of this technology include non-contact, high speed, and so forth. In the early stage, many literatures studied the laser interaction on the polyimide surface [6,7,8]. The flexibility, controllability, simplicity, and low-cost of laser-induced graphene over conventional methods has rapidly attracted the attention of manufacturers to employ it in the fabrication of high-performance supercapacitors [9,10,11], gas sensors [12,13], strain sensors [14,15], antibacterial devices [16,17], and temperature sensors [18]

A variety of materials have been converted into LIG by using a 10.6 μm CO_2_ laser, ranging from paper, cloth, to food [19,20,21]. Furthermore, various lasers with different wavelengths have been used to induce LIG. For example, a 355 nm pulsed laser and 450 nm pulsed laser were used to convert PI into LIG, and the produced LIG were applied to strain sensors and all-solid-state planar integrated micro-supercapacitors [22]. Polydimethylsiloxane (PDMS) was converted into LIG by using a continuous mode diode laser with a wavelength of 405 nm [23]. LIG formed on PI by using a 405 nm ultraviolet (UV) laser was applied to gas sensors [13]. However, there is a lack of understanding of how different lasers (wavelengths) perform differently in the LIG conversion process. 

After emerging LIG by using a CO_2_ laser, researchers attempted to apply other types of lasers, including a UV laser, visible laser, and ultra-short pulse laser to generate LIG. They evaluated the transition process by investigating the photothermal and the photochemical effects. The photothermal effects tended to occur in lasers with long wavelengths. Thus, the LIG formed on PI by using CO_2_ laser was more likely to be governed by the photothermal effect [8]. In the case of the 355 nm UV laser, before the discovery of LIG, many papers studied the mechanism of UV laser ablation of PI. Yung et al. [24] reported that a photothermal mechanism contributed to the ablation process. Shin et al. [25] noted that photothermal and photochemical reactions exist simultaneously. It was found that the laser ablation of PI was dominated by the photochemical effect at the low laser fluence, while at the high laser fluence, it was governed by the photothermal effect. In a study conducted by Du et al. [26], it was indicated that the UV laser ablation process was probably dominated by the photochemical effect, while for the 1064 nm laser, the ablation was governed by the photothermal effect. However, it was not reported that the surface morphology was analyzed to determine whether LIG formation was influenced by the photothermal or photochemical effect.

In this paper, a 10.6 μm CO_2_ infrared laser and a 355 nm UV laser are employed to produce LIG from PI. The produced LIGs are compared from surface morphology, atomic content, sheet resistance, and Raman spectrum. Meanwhile, the ablation effects are also investigated.

## 2. Materials and Methods 

In this study, two types of lasers, i.e., a 40 W CO_2_ infrared laser (wavelength of 10.6 μm) and a 5 W UV laser (wavelength of 355 nm), were used for fabricating LIG from PI. The repetition rate of CO_2_ laser ranged from 0 to 15 kHz, the scan rate was from 0 to 2000 mm/s. The repetition rate of 12 kHz, line spacing of 10 μm, and scan rate of 100 were used for the experiments. The UV laser also provided some options of controlling the repetition rates from 20 to 200 kHz and the scan rate from 0 to 2000 mm/s. In order to obtain the same laser fluence as the CO_2_ laser, the repetition rates of 20 kHz were used for experiments. The UV laser with a low scan rate and small line spacing would destroy the substrate. Therefore, in order to get a complete substrate for the subsequent characterization, a scan rate of 300 mm/s and line spacing of 20 μm were used for the experiment. In the experiments, it was found that the change of laser fluence had a great effect on the PI surface, and both the CO_2_ laser and UV laser used laser fluence from 4.3 J/cm^2^ to 12 J/cm^2^ for the comparative experiment. In addition, argon (Ar) was used to check the effects of the atmosphere on the element content and compare with ambient conditions, all the rest of the experiments were performed under ambient conditions. The properties of the studied substrates are indicated in Table S1. 

The laser beams were delivered to the substrate by a set of galvanometer scanners and focusing optics for both lasers. The focal length was 280 mm for the CO_2_ laser and 295 mm for the UV laser, respectively. The focal spot size (assuming a Gaussian beam distribution) was calculated to be 200 μm and 40 μm for the CO_2_ laser and UV laser, respectively. The process parameters for the laser treating PI were defined in Table 1. The line spacing was defined as the central distance between the two adjacent lines, which controls the scanned area. The PI sheet was locally cooled with a layer of water underneath the PI substrate to reduce PI substrate deformation due to laser-induced heating.

The sheet resistance was measured by a four-point probe instrument in a range of 10^−2^ Ω/square to 10^5^ Ω/square with probe spacing of 1 mm. Additionally, scanning electron microscopy (SEM) (Quanta 250 FEG, accelerating voltage: 0.2–30 kW, Thermo Fisher Scientific, 81 Wyman Street, Waltham, MA, USA) was used to analyze morphologies of the LIG surfaces. The material properties of the PI surface were measured by Raman spectroscopy (HR Evolution, spectrum range: 200–2100 nm, Horiba Scientific, France). Furthermore, the three-dimensional morphologies of LIG were measured by a 3D profilometer (UP-Dule Mode, Rtec Instrument, 1810 Oakland Rd, B San Jose, CA, USA). 

## 3. Results and Discussion

### 3.1. Laser-Induced Graphene

**Figure 1:**
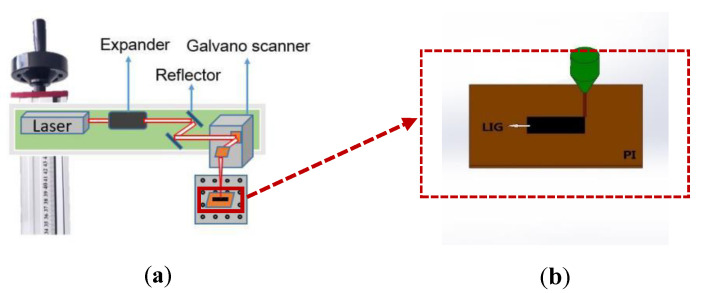
Schematic diagrams of (**a**) the laser scanning process and (**b**) the laser-induced graphene (LIG) conversion process.

The schematic laser-induced graphene process is shown in Figure 1, where a set of galvanometers are used to control the beam motion over the PI substrate. Figure 1a shows the laser optics delivery system and Figure 1b shows the laser-processed area on the substrate.

### 3.2. Optical Performance Analysis

**Figure 2:**
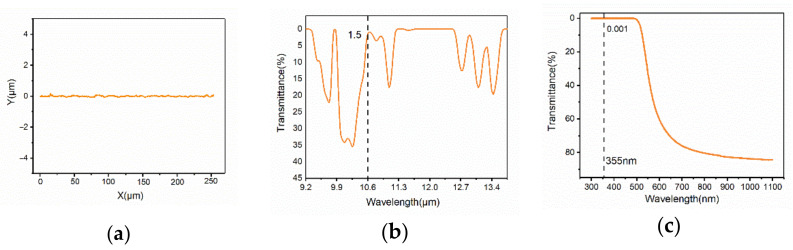

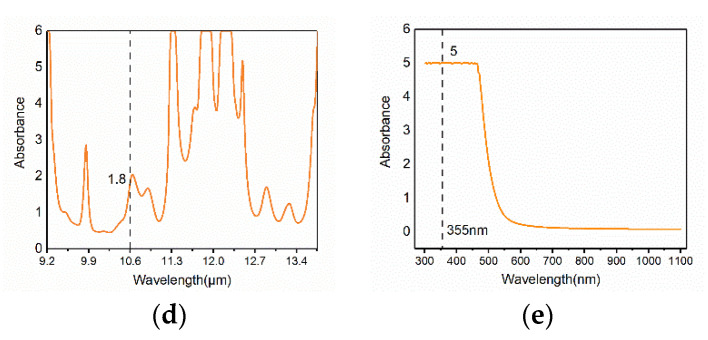
(**a**) Surface profile of pristine polyimide (PI); (**b**) infrared and UV-vis (**c**) transmission spectrums of pristine PI film; infrared (**d**) and UV-vis (**e**) absorption spectrums of pristine PI film.

The pristine PI surface profile is shown in Figure 2a, where it can be seen that the pristine PI surface is smooth. The infrared and UV-vis transmission spectrums of pristine PI film are shown in Figure 2b,c, which indicates that the PI film has a weak transmission at both 355 nm and 10.6 μm. Figure 2d,e shows the infrared and UV-vis absorption spectrums of pristine PI film, which demonstrates that PI has intensive absorption for both UV laser and CO_2_ laser. The SEM image of the pristine PI surface is presented in Figure S1.

### 3.3. Morphological Characterization

**Figure 3:**
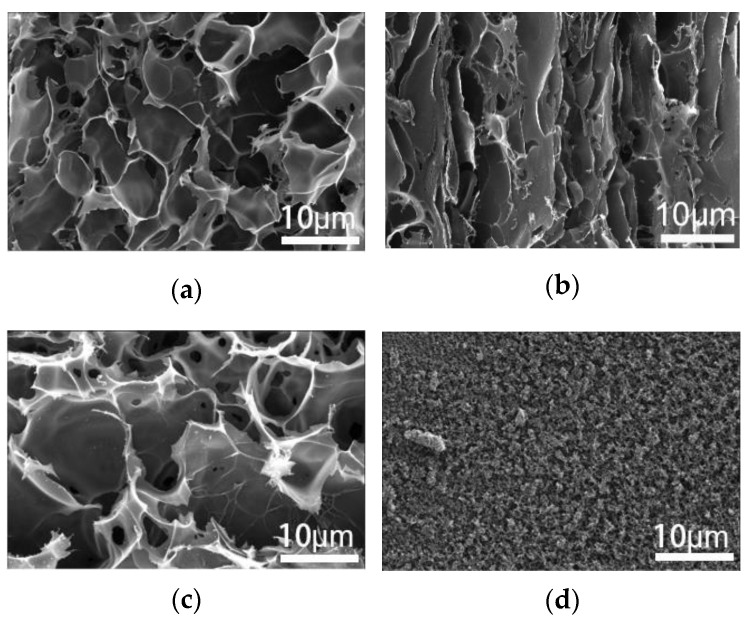
Scanning electron microscopy (SEM) images of surface-treated by CO_2_ laser with a laser fluence of (**a**) 6.6 J/cm^2^ and (**b**) 12 J/cm^2^; SEM images of surface-treated by UV laser with a laser fluence of (**c**) 6.6 J/cm^2^ and (**d**) 12 J/cm^2^.

The SEM images of the treated surface under the two lasers and different processing conditions are shown in Figure 3. The PI surface treated by a CO_2_ laser with a laser fluence of 6.6 J/cm^2^ appears to have a porous structure with the pore sizes ranging from 4 to 8 microns, as depicted in Figure 3a. The porous surface structure is likely caused by the escape of gases, due to the local high temperature. When the CO_2_ laser fluence increased to 12 J/cm^2^, the PI surface had a surface morphology with fewer pores (Figure 3b). The main reason is that excessive energy burned off the edge of the pores and thus closed up the pores. The UV laser-treated surface shows two different porous morphologies, as shown in Figure 3c. When the UV laser fluence was 6.6 J/cm^2^, the induced surface morphology was similar to that induced by the CO_2_ laser shown in Figure 3a. The pore radius was much smaller in the nanometer scale with a UV laser fluence of 12 J/cm^2^ (Figure 3d). The reason for this phenomenon is that the ablative area rapidly vaporizes to form nanometer-sized pores at the higher laser fluence.

**Figure 4:**
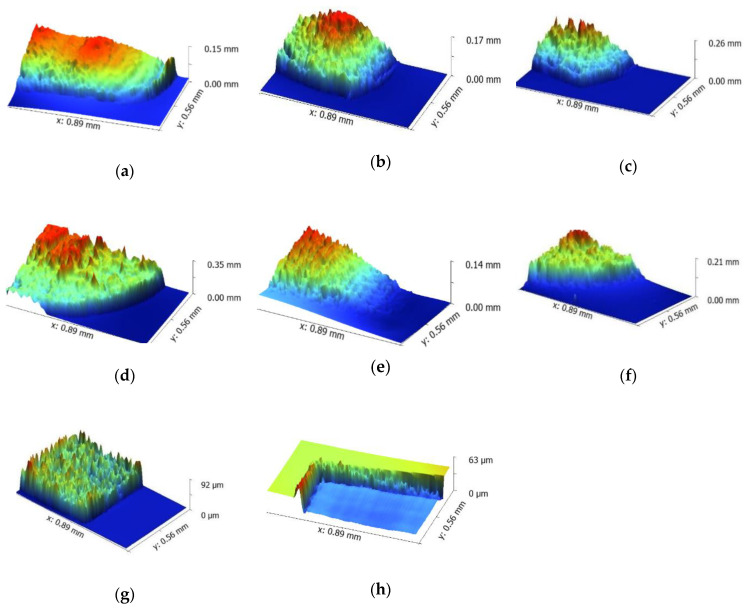
The surface morphology of the edge of the treated zone measured by the 3D profilometer. Raised structure formed on PI by using CO_2_ laser with a laser fluence of (**a**) 6.6 J/cm^2^, (**b**) 8.2 J/cm^2^, (**c**) 10.4 J/cm^2^,and (**d**) 12 J/cm^2^; raised structure formed on PI by using UV laser with a laser fluence of (**e**) 6.6 J/cm^2^, (**f**) 8.2 J/cm^2^ (**g**) 10.4 J/cm^2^; concave structure formed on PI by using a UV laser with a laser fluence of (**h**) 12 J/cm^2^.

The absorption of 10.6 μm laser by PI raised substrate temperatures, leading to a raised and inflated structure (Figure 4a–d). Clear traces of melting around the processing region was also observed. Hence, the CO_2_ laser interaction with PI is likely governed by a photothermal mechanism. After the UV laser irradiation, as seen in Figure 4e–g, obvious photothermal features, such as raised and inflated structure and traces of melting, appeared in the ablative region. When the UV laser fluence was at 12 J/cm^2^, the concave structure appeared on the surface (Figure 4h), due to the higher laser fluence. There were no obvious traces of melting around the processing region. The formation of such a concave structure is dominated by the removal of material. 

### 3.4. Chemical Composition Analysis

**Figure 5:**
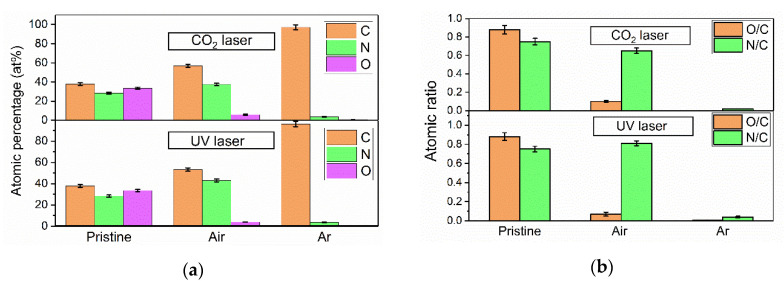
The atomic content measured by energy dispersive spectrometer (EDS); (**a**) atomic content in a variety of environments; (**b**) atomic ratio in a variety of environments.

The energy dispersive spectrometer (EDS) was used to evaluate the trend in surface atomic content variation. The results from the EDS are semi-quantitative and they are used to highlight trends in the variation of the chemical composition but not to accurately measure the chemical composition. The atomic weight percentages of carbon (C), nitrogen (N), and oxygen (O) are shown in Figure 5a. The carbon content increased significantly after laser treatment. Through data calculation, the O-to-C ratio and N-to-C ratio are displayed in Figure 5b. Compared to the pristine PI, whether in air or in Ar, the O-to-C atomic ratio decreased when the PI was processed with 10.6 μm or 355 nm laser, and the N-to-C atomic ratio decreased with 10.6 μm laser ablation. The N-to-C atomic ratio decreased with UV laser ablation in the Ar protection environment. The cause for this phenomenon is that the high temperatures from laser irradiation broke the C-N bonds and C-O bonds, both oxygen and nitrogen atomic contained with elements in the atmosphere to form gaseous products, such as O_2_, N_2_, CO, CH_4_, and so forth [27]. Meanwhile, the PI surface began to carbonize [28] and formed a graphene structure. It is worth noting that the N-to-C atomic ratio increased in the air with 355 nm laser ablation, which indicates that the reaction involves the addition of nitrogen from the air. It should be noted that the nitrogen molecules in air were ionized by the high photon energy and high peak power of the 355 nm beam, and the ionized nitrogen element could have reacted with the active groups on the PI surface. This process involves the photochemical effect [26]. 

The single-photon energy of the 355 nm UV laser was 337 kJ/mol, which would break the C-C bonds (332 kJ/mol), C-O bonds (326 kJ/mol), and C-N bonds (305 kJ/mol). However, the single-photon energy of the 10.6 μm CO_2_ laser was only 11.3 kJ/mol, with which it was virtually impossible to break the chemical bonds by single-photon absorption. The energy from CO_2_ laser irradiation triggered the lattice vibration, which gave rise to such high temperatures, leading to the C-O and C-N bonds being broken by this temperature. Compared to 10.6 μm CO_2_ laser, a shorter wavelength and high fluence UV laser is more likely to induce photochemical reactions.

**Figure 6:**
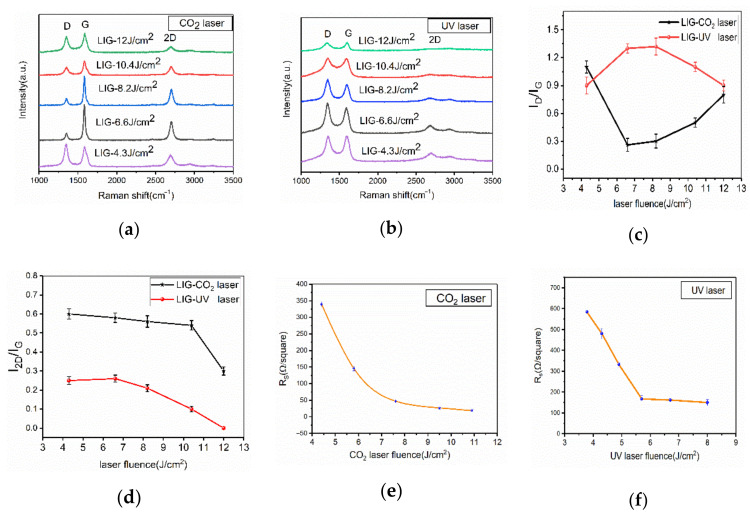
LIG formed on PI using CO_2_ laser and UV laser; (**a**) representative Raman spectrum of LIG formed by UV laser; (**b**) representative Raman spectrum of LIG formed by CO_2_ laser; (**c**) statistical analysis of the ratios of the D and G peak intensities (I_D_/I_G_); (**d**) statistical analysis of the ratios of the 2D and G peak intensities (I_2D_/I_G_); sheet resistance (R_s_) of LIG at various (**e**) CO_2_ laser and (**f**) UV laser energy fluences.

The Raman spectra of the laser-treated PI surface are shown in Figure 6. There are three significant peaks: D, G, and 2D, which describe the characteristics of the laser-induced graphene. The D peak at 1350 cm^−1^ is used to characterize defects or edges of the LIG. The G peak at 1580 cm^−1^ and the 2D peak at 2700 cm^−1^ indicate the number of LIG layers. The 2D peak is used to verify that the material is graphene. The ratio of D to G indicates the disorder, crystallinity, and defects of the formed graphene [4,8,15]. After CO_2_ laser irradiation, the representative Raman spectrum shows a higher 2D peak (Figure 6a), implying that a few-layered graphene has been generated on the PI surface, as shown in a previous study [23]. Figure 6a shows the representative Raman spectrum of LIG formed by the UV laser, a lower 2D peak demonstrates the graphene’s graphene-like flake structure, as shown in a previous study on LIG [21]. As shown in Figure 6c, with the CO_2_ laser treatment, the I_D_/I_G_ ratio shows an earlier decrease and later increase trend, this decrease trend is due to an increase in surface temperature, and this increase trend is attributed to the oxidation in ambient condition [4]. The I_D_/I_G_ ratio showed an opposite trend under the UV laser. From 4.3 J/cm^2^ to 8.2 J/cm^2^, the I_D_/I_G_ ratio shows the same increase trend as in a previous study [15], which is attributed to high UV laser single-photon energy. Subsequently, as the laser energy increased, it was dominated by material removal, the surface of PI produced a nanostructure that was different from LIG, which shows a low I_D_/I_G_ ratio. The higher I_D_/I_G_ ratio implies more LIG defects and lower crystallinity. Garland et al. [13] explained that UV laser beams exhibit lower absorption of PI, and thus, the product was a mixture of graphene and amorphous carbon. However, in our study, as shown in Figure 2, it shows that PI has an intensive absorption and weak transmittance for both UV laser and CO_2_ laser. Figure 6d shows that the I_2D_/I_G_ ratio in LIG induced by the UV laser is lower than that induced by the CO_2_ laser, which indicates that the LIG treated by CO_2_ laser have fewer layers.

Figure 6e shows the sheet resistance of LIG at various CO_2_ laser powers. The original sheet resistance of the PI was more than 90 MΩ per square, which acted as a good insulator. However, it became electrically conductive after the laser treatment. It was experimentally found that the threshold for the sheet resistance reduction was at the laser energy fluence of 4.4 J/cm^2^ and the scanning speed of 100 mm/s. As is depicted in Figure 6e, the sheet resistance decreased from 340 to 20 Ω per square with the increase in laser fluence, probably due to the increase in the degree of graphitization. The high electrical conductivity of the LIG would have facilitated the practical applications in the manufacture of the sensors. Figure 6f shows the sheet resistance of LIG formed on PI using the UV laser at various levels of energy fluence, the sheet resistance decreased with the increase of the laser energy fluence and dropped to a level of 160 Ω per square at 5.5 J/cm^2^ and remained almost constant with a further increase of the energy fluence to 8 J/cm^2^. Under the same laser fluence, the LIG induced by the CO_2_ laser had a much lower sheet resistance at 20 Ω per square. 

## 4. Conclusions

In this article, differences in the LIGs formed under the two types of lasers (CO_2_ infrared laser and 355 nm UV laser) on a locally water-cooled PI substrate were investigated from four aspects, i.e., surface morphology, atomic weight percentages, sheet resistance, and Raman spectrum. It is shown that PI was converted into graphene by both the CO_2_ and the UV lasers. The LIG conversion process by both lasers was principally due to the photothermal effect, and the photochemical effect was also involved in the UV laser LIG conversion. The surfaces treated by the CO_2_ laser were characterized by micron-sized pores and a sheet structure with fewer pores, whereas the surfaces treated by UV laser displayed micron-sized and nanometer pores. From the trend in atomic content variation, the 355 nm UV laser treated PI surface showed an increase of nitrogen atoms, whereas the CO_2_ laser treated surface showed a decrease in the nitrogen atoms. From the Raman spectrum, the 2D-to-G ratio and 2D peak indicate that LIG formed by CO_2_ laser possessed better quality and had fewer graphene layers. Therefore, the CO_2_ laser is a preferred wavelength for LIG on the PI substrate.

## Figures and Tables

**Table 1:** The employed process parameters for treating PI.

	CO_2_ Laser	UV Laser
Scan rate	100 mm/s	300 mm/s
Repetition rates	12 kHz	20 kHz
Line spacing	10 μm	20 μm

## Supplementary Materials

The following are available online at https://www.mdpi.com/2072-666X/11/12/1094/s1, Figure S1: The SEM image of the pristine PI surface, Table S1: The main properties of PI.
