# Supplementary material for: A Comparative Study of Laser-Induced Graphene by CO2 Infrared Laser and 355 nm Ultraviolet (UV) Laser
Source: Micromachines (Basel). 2020 Dec 11;11(12):1094. doi: 10.3390/mi11121094 (PMC7764730; doi:10.3390/mi11121094)
Supplement: Supplementary file 1 [file micromachines-11-01094-s001.pdf]

micromachines

Supplementary Materials

A comparative study of laser-induced graphene by CO2 infrared laser and 355 nm Ultraviolet (UV) laser

Liyong WANG [1], Zhiwen WANG [1], Ali Naderi Bakhtiyari [1] and Hongyu ZHENG [1,*]

[1] Centre for Advanced Laser Manufacturing (CALM), School of Mechanical Engineering, Shandong University of Technology, Zibo 255000, Shandong
* Correspondence: zhenghongyu@sdut.edu.cn; Tel.: +86-533-2780169

Table S1. The main properties of the PI

Substrate	Type	Thickness	Thermal decomposition temperature	Relative density	Tensile strength
PI	Thermoset	0.125 mm	550℃	1.4	200 MPa

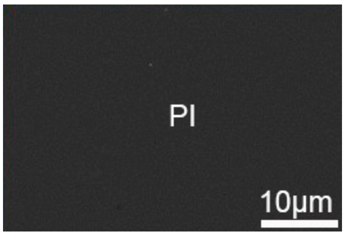

Figure S1. The SEM image of the pristine PI surface

Publisher's Note: MDPI stays neutral with regard to jurisdictional claims in published maps and institutional affiliations.

© 2020 by the authors. Submitted for possible open access publication under the terms and conditions of the Creative Commons Attribution (CC BY) license (http://creativecommons.org/licenses/by/4.0/).
